# Polarization Dependence Suppression of Optical Fiber Grating Sensor in a π-Shifted Sagnac Loop Interferometer

**DOI:** 10.3390/s100504373

**Published:** 2010-04-29

**Authors:** Jaebum Son, Min-Kyoung Lee, Myung Yung Jeong, Chang-Seok Kim

**Affiliations:** 1 School of Medicine, Korea University, Seoul, 136-705, Korea; E-Mail: jaebum.son@gmail.com; 2 Department of Physics, Chungnam National University, Daejeon, 305-764, Korea; E-Mail: leemk@keit.re.kr; 3 W.C.U. Department of Cogno-Mechatronics, Pusan National University, Busan, 609-735, Korea; E-Mail: myjeong@pusan.ac.kr

**Keywords:** fiber sensor, long-period fiber gratings, sagnac effect, polarization-sensitive devices

## Abstract

In the sensing applications of optical fiber grating, it is necessary to reduce the transmission-type polarization dependence to isolate the sensing parameter. It is experimentally shown that the polarization-dependent spectrum of acousto-optic long-period fiber grating sensors can be suppressed in the transmission port of a π-shifted Sagnac loop interferometer. General expressions for the transmittance and reflectance are derived for transmission-type, reflection-type, and partially reflecting/transmitting-type polarization-dependent optical devices. The compensation of polarization dependence through the counter propagation in the Sagnac loop interferometer is quantitatively measured for a commercial in-line polarizer and an acousto-optic long-period fiber grating sensor.

## 1. Introduction

Polarization-dependence loss (PDL) of transmission-type optical devices, such as long-period fiber grating (LPFG) sensor and wavelength-tunable filters, is an inherent problem that limits information capacity in optical sensor applications [1,2]. In additional to the transmission-type LPFG sensor, the fiber Bragg grating (FBG) sensor, which is a partially transmission/reflection-type fiber device, also suffers from PDL [3]. A simple compensation method to suppress the PDL of transmission-type fiber gratings using a Sagnac loop interferometer has been recently demonstrated [4,5]. However, the polarization dependence of the Sagnac interferometer containing other types of devices has yet to be addressed.

In this work, three types of polarization-dependent devices, a transmission-type, reflection-type, and mixed (both transmitting and reflecting)-type, are analyzed by use of Jones matrix method. It is shown that the polarization-dependent spectra of transmission-type optical devices can be suppressed in the transmission port of π-shifted Sagnac loop interferometer [5,6], and that the PDL of reflection-type optical devices can be suppressed in the reflection port of π-shifted Sagnac loop interferometer. An analysis of the PDL of a Sagnac interferometer containing a mixed-type device is also performed.

We also characterize experimentally PDL suppression using Sagnac loop interferometer for various devices exhibiting various degrees of PDL such as an in-line polarizer (ILP) and an acousto-optic LPFG sensor. The cancellation of polarization dependence through the counter propagation in the Sagnac loop interferometer is quantitatively measured using a commericial PDL measurement control system. This simple PDL suppression method can be used to minimize the various PDL effect.

## 2. Theoretical Analysis

**Figure 1.**
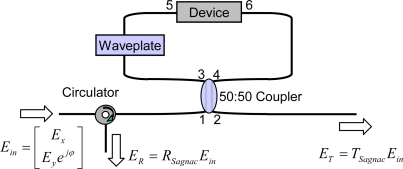
Schematic diagram of the Sagnac interferometer with a polarization-dependent device and a waveplate inside the loop.

A schematic diagram of the Sagnac loop interferometer with a polarization-dependent device, similar to the one demonstrated in [5], is shown in Figure 1. In this work, we also consider the cases when the polarization-dependent device is a reflection-type and mixed-type device, as well as the previous described transmission-type device, as shown in Table 1. Assuming that the principal polarization axes of the device are along the x and y axes of laboratory coordinates [7], the polarization-dependent transmission, *T_device_*, between the input and transmitted field and the reflection, *R_device_*, between the input field and reflected field are described by the Jones matrices in Table 1. In these matrices, amplitude factors *α*, *β*, *δ*, and *ε* are all real numbers and birefringence of the device is considered to be negligible. These transmission and reflection relations hold for bi-directional propagation.

Using a procedure similar to the one shown in [5], the transmittance, *T_Sagnac_*, and reflectance, *R_Sagnac_*, of a Sagnac interferometer can be solved analytically using Jones matrix analysis. In the waveplate in Figure 1, two parameters are considered; a retardance, Γ, and an orientation of waveplate axes, *θ*, with respect to the laboratory coordinates. (We should consider the general case.) The two cases when *θ* = 0, Γ = 0 and *θ* = π/4, Γ = π are expressed in Tables 2 and 3, respectively. For the waveplate with *θ* = 0, Γ = 0 (no waveplate), the Sagnac loop interferometer works as mirror; that is, it exhibits maximum reflection and minimum transmission. Contrarily, for a waveplate with *θ* = π/4, Γ = π (π-shifted waveplate at angle π/4), maximum transmission and minimum reflection can be achieved in Ports 2 and 1, respectively. We consider Port 1 as the reflection port and Port 2 as the transmission port of a Sagnac interferometer with a nested optical device. With *l_1_* set equal to the length between Ports 3 and 5 and *l_2_* set equal to the arm length between between Ports 4 and 6, *Δl* is defined as *l_1_* − *l_2_*. When *Δl* = 0 or *l_1_* = *l_2_*, as the Sagnac interferometer is referred to as a length-symmetric Sagnac loop interferometer. The longitudinal phase constant for a traveling wave in the fiber is represented as *k*.

Table 2 shows that the reflectance, *R_Sagnac_*, contains polarization-dependent terms in each of the three types of devices. The transmittance, *T_Sagnac_*, also includes polarization-dependent terms, except for the case of a transmission-type device, in which the intensity is null at the transmission port. In comparison, the π-shifted Sagnac loop interferometer shows us an interesting theoretical result. Both the reflectance, *R_Sagnac_*, and the transmittance, *T_Sagnac_*, do not have polarization-dependent terms in both transmission-type and reflection-type devices. The case of Equation (13) has been previously described in [5] as a specific example in which the polarization-dependent transmission of a transmission-type device is clearly averaged through the use of a π-shifted Sagnac loop interferometer configuration [5].

In this research, more general cases are considered. For a length-symmetric Sagnac loop interferometer, the expression for the reflectance in Equation (16) can be further simplified to

(16-1) $$1/4{\left(\delta +\varepsilon \right)}^{2}$$

meaning that the polarization-dependent reflection of a reflection-type device can also be compensated.

## 3. Experiment with In-Line Polarizer

As a representative polarization-dependent device having the highest polarization dependence to the input polarization state, a commercial in-line polarizer (ILP-155-09-09-1) is selected in this experiment [4] because the quantitative proof of this suppression effect has not been sufficiently investigated using commercially available measurement tools. In this work, we measure the PDL value of a commercial in-line polarizer using a commercial PDL measurement control system without and with a Sagnac loop configuration, respectively.

**Figure 2.**
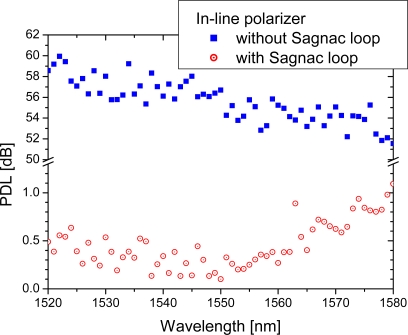
For each configuration of in-line polarizer without and with a Sagnac loop, the average PDL is measured to be 55.6 dB and 0.45 dB, respectively.

Without a Sagnac loop, the average PDL of the ILP is measured to be ∼55 dB in each transmission direction. However, the average PDL value is dramatically reduced to less than 0.45 dB when it is placed within a Sagnac loop. The polarization dependence, quantified as PDL, can be measured from a control system consisting of a tunable laser (Ando, AQ4321B), polarizer, and polarimeter (Tektronix, PAT9000B). The intrinsic polarization dependence of the transmission-type polarization-dependent optical device can be measured by placing it between polarizer and polarimeter in any direction when the device has similar transmission characteristics in both transmission directions. Thus, the measured PDL values are similarly measured in both directions. As shown in Figure 2, the PDL was measured to have very low values below 0.5 dB around 1,550 nm, but slightly increased as the wavelength moved over 1,560 nm. We believe that this is caused by the wavelength dependence of intensity splitting ratio of 50:50 coupler. In general, it has been known that the C-band 50:50 coupler has the optimal splitting ratio centered around 1,550 nm.

These can in principle be minimized by optimizing all components used to construct the Sagnac loop. A large number of the packaged optical devices have been treated as failure products at their final test stage just due to the high PDL value only. It is expected that the intrinsic and fabrication-induced PDL of these products can be easily compensated with the incorporation of a Sagnac loop in the packaging process.

## 4. Experiment with Acousto-Optic LPFG Sensor

The polarization dependent transmission and reflection of an acousto-optic LPFG sensor are also experimentally examined by placing it inside the Sagnac loop interferometer with waveplate and are compared with expressions in Equations (13) and (8), respectively. Since acousto-optic LPFG sensors and micro-bending LPFGs are functionally based on asymmetrical cladding mode coupling, these transmission-type devices show intrinsically polarization-dependent transmission spectra and no back reflection [10,11]. Spectrally-resolved and polarization-dependent coupling to each of the cladding TE_01_, TM_01_, and HE_21_ modes is clearly observed for an acousto-optic LPFG sensor with an interaction region consisting of a 28-cm-long dispersion compensating fiber (OFS EHS100), [8]. The input polarization state is varied using a polarized broadband source and an all-fiber polarization controller [9]. The output spectra at transmission and reflection ports are measured using an optical spectrum analyzer in order to analyze the spectral PDL.

**Figure 3.**
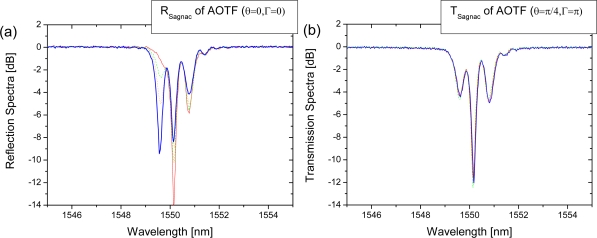
For various input polarization states, optical spectra of polarization-dependent acousto-optic LPFG sensor in a Sagnac loop are measured from (a) the reflection port of Sagnac loop (*θ* = 0, Γ = 0) and (b) the transmission port of Sagnac loop (*θ* = π/4, Γ = π).

For the case of Equation (8), the waveplate is tuned to maximize the reflected output and minimize the transmission output (Γ = 0 and *θ* = 0). The spectral reflectance is easily changed by tuning the input polarization as shown in Figure 3 (a). The filtering spectra for four different input polarization states are shown in each figure. It clearly shows that the intrinsic polarization-dependence of the transmission of an acousto-optic LPFG sensor is transferred to the spectrum measured at the reflection port of the Sagnac loop mirror. In contrast, the polarization-dependent mode splitting is completely eliminated when the waveplate settings are adjusted so that Γ = π and *θ =* π/4 by maximizing the intensity in the transmission Port 2 as shown in Figure 3 (b) and derived in Equation (13).

## 4. Conclusions

We have generally analyzed and measured the polarization dependence cancellation of optical fiber grating sensor using a π-shifted Sagnac loop interferometer. For the feasible intrinsic PDL-free integrated optical devices, a commercial-grade in-line polarizer device was tested to verify the proposed PDL compensating method. A representative type of polarization-dependent device, an acousto-optic LPFG sensor, is also experimentally proved for the polarization-dependence suppression. It is expected that the use of the Sagnac loop can save many optical sensor products, such as FBG sensors and micro-bending LPFG sensors which have been limited in their applications solely due to their high PDL.

## Figures and Tables

**Table 1.** Transmission and Reflection of each type of PDL device are presented in Jones matrix form.

**Withtout Sagnac interferometer**		
Transmission-type polarization dependent device	*T_Device_*	(1) $$\left[\begin{array}{cc} \alpha & 0\\ 0 & \beta \end{array}\right]$$
	*R_Device_*	(2) $$\left[\begin{array}{cc} 0 & 0\\ 0 & 0 \end{array}\right]$$
Reflection-type polarization dependent device	*T_Device_*	(3) $$\left[\begin{array}{cc} 0 & 0\\ 0 & 0 \end{array}\right]$$
	*R_Device_*	(4) $$\left[\begin{array}{cc} \delta & 0\\ 0 & \varepsilon \end{array}\right]$$
Mixed-type polarization dependent device	*T_Device_*	(5) $$\left[\begin{array}{cc} \alpha & 0\\ 0 & \beta \end{array}\right]$$
	*R_Device_*	(6) $$\left[\begin{array}{cc} \delta & 0\\ 0 & \varepsilon \end{array}\right]$$

**Table 2.** The transmittance and reflectance of a Sagnac interferometer with various nested devices exhibiting PDL when the waveplate settings are *θ* = 0 and Γ = 0.

**With the Sagnac loop of *θ* = 0, Γ = 0**		
Transmission-type polarization dependent device	*T_Sagnac_*	0 (7)
	*R_Sagnac_*	(8) $\left(E_{x}^{2}\alpha^{2}+E_{y}^{2}\beta^{2}\right)/\left(E_{x}^{2}+E_{y}^{2}\right)$
Reflection-type polarization dependent device	*T_Sagnac_*	(9) $\frac{1}{E_{x}^{2}+E_{y}^{2}}\left(E_{x}^{2}\delta^{2}+E_{y}^{2}\varepsilon^{2}\right)\;{\text{cos}}^{2}\left(k\Delta l\right)$
	*R_Sagnac_*	(10) $\frac{1}{E_{x}^{2}+E_{y}^{2}}\left(E_{x}^{2}\delta^{2}+E_{y}^{2}\varepsilon^{2}\right)\;{\text{sin}}^{2}\left(k\Delta l\right)$
Mixed-type polarization dependent device	*T_Sagnac_*	(11) $\frac{1}{E_{x}^{2}+E_{y}^{2}}\left(E_{x}^{2}\delta^{2}+E_{y}^{2}\varepsilon^{2}\right){\text{sin}}^{2}\left(k\Delta l\right)$
	*R_Sagnac_*	(12) $$\begin{array}{l} \frac{1}{2\left(E_{x}^{2}+E_{y}^{2}\right)}\left(E_{x}^{2}\left({2\alpha }^{2}+\delta^{2}\right)+E_{y}^{2}\left({2\beta }^{2}+\varepsilon^{2}\right)\right)\\ -\left(E_{x}^{2}\delta^{2}+E_{y}^{2}\varepsilon^{2}\right)\text{cos}\left(2k\Delta l\right)\\ +\left(-4E_{x}^{2}\alpha \delta +4E_{y}^{2}\beta \varepsilon \right)\text{sin}\left(k\Delta l\right) \end{array}$$

**Table 3.** The transmittance and reflectance of a Sagnac interferometer with various nested devices exhibiting PDL when the waveplate settings are *θ* = π/4 and Γ = π.

**With the Sagnac loop of *θ* =π/4, Γ = π**		
Transmission-type polarization dependent device	*T_Sagnac_*	(13) $\frac{1}{4}{\left(\alpha +\beta \right)}^{2}$
	*R_Sagnac_*	(14) $\frac{1}{4}{\left(\alpha -\beta \right)}^{2}$
Reflection-type polarization dependent device	*T_Sagnac_*	(15) $\frac{1}{4}(\delta^{2}+\varepsilon^{2}-2\delta \varepsilon \;\text{cos}(2k\Delta l))$
	*R_Sagnac_*	(16) $\frac{1}{4}\left(\delta^{2}+\varepsilon^{2}+2\delta \varepsilon \;\text{cos}\left(2k\Delta l\right)\right)$
Mixed-type polarization dependent device	*T_Sagnac_*	(17) $$\begin{array}{l} \frac{1}{4}{[\left(\alpha +\beta \right)}^{2}+\delta^{2}+\varepsilon^{2}-2\delta \varepsilon \;\text{cos}\left(2k\Delta l\right)\\ +4\frac{E_{x}E_{y}}{E_{x}^{2}+E_{y}^{2}}\left(\alpha +\beta \right)(-\delta \;\text{cos}\left(k\Delta l-\phi \right)\\ +\varepsilon \;\text{cos}\left(k\Delta l+\phi \right))] \end{array}$$
	*R_Sagnac_*	(18) $$\begin{array}{l} \frac{1}{4}{[\left(\alpha -\beta \right)}^{2}+\delta^{2}+\varepsilon^{2}+2\delta \varepsilon \;\text{cos}\left(2k\Delta l\right)\\ -4\frac{E_{x}E_{y}}{E_{x}^{2}+E_{y}^{2}}\left(\alpha -\beta \right)(\delta \;\text{cos}\left(k\Delta l-\phi \right)\\ +\varepsilon \;\text{cos}\left(k\Delta l+\phi \right))] \end{array}$$
